# KIR diversity in three ethnic minority populations in China

**DOI:** 10.1186/s12967-015-0544-7

**Published:** 2015-07-11

**Authors:** Qiongxiu Zhou, Jue Wang, Zhi He, Xiaojuan Li, Song Mao, Shu Huang, Guohui Bian, Feng Ma

**Affiliations:** Center for Stem Cell Research and Application, Institute of Blood Transfusion, Chinese Academy of Medical Sciences and Peking Union Medical College (CAMS and PUMC), 26 Huacai Road, Longtan Industry Park, Chenghua District, Chengdu, 610052 China; Urumqi Blood Center, Urumqi, China; Tibet Blood Center, Lhasa, China; Sichuan Cord Blood Bank, Chengdu, China

**Keywords:** KIR gene, Genotype, Haplotype, Ethnic minority populations in China

## Abstract

**Background:**

Killer cell immunoglobulin-like receptors (KIRs) show extensive variation in genetic content and allelic polymorphi sms among different populations.

**Materials and methods:**

We analyzed the distribution of KIR genes in the Tibetan ethnic minority of Lhasa city, the Uyghur and Kazakh ethnic minorities of Urumqi city populations in China. Genotyping of 16 KIR genes was tested in 479 randomly selected individuals using the multiple PCR-SSP method.

**Results:**

A total of 42 KIR genotypes were detected, of which, 29 were predicted to be AB genotypes, 12 were BB genotypes and one was AA genotypes. 27 KIR genotypes were identified in Kazakhs, 30 KIR genotypes were identified in Uyghurs and 20 KIR genotypes were identified in Tibetans. The predominant genotype 1(AA genotypes) occurred most frequently in Tibetans (52.7%, 118/224), Kazakhs (43.2%, 54/125) and Uyghurs (34.9%, 45/130). Not only the four framework genes were present in all individuals, but the pseudogene *2DP1* could also be detected in all Uyghur individuals. Tibetans were different from Kazakh and Uyghur groups in KIR genetic content and *KIR* allelic variation. Intriguingly, Tibetans (29.5%, 66/224) had lower frequencies of *2DS4*-*v* when compared with Uyghurs (60.8%, 79/130) and Kazakh s (59.2%, 74/125). Uyghurs (25.4%, 33/130) displayed higher frequencies of Bx genotypes with C4Tx (absence of *KIR3DS1*-*2DL5*-*2DS5*-*2DS1*) than both Kazakhs (11.2%, 14/125) and Tibetans (3.6%, 8/224).

**Conclusions:**

The study showed that profile of KIR genotypes in three ethnic minority populations in China displayed ethnic diversity. It could be valuable for enriching the ethnical information resources for KIR gene, as well as facilitating further research on KIR-related diseases.

**Electronic supplementary material:**

The online version of this article (doi:10.1186/s12967-015-0544-7) contains supplementary material, which is available to authorized users.

## Background

Killer cell immunoglobulin-like receptors (KIRs) are a group of glycoproteins expressed on the surface of Natural killer (NK) cells [1, 2]. NK cells are important components of the innate immune system that take part in various immune responses to different infectious agents [3].

KIR genes are located on human chromosome 19q13.4. Up to now, 16 KIR gene loci have been identified, including two pseudogenes (KIR *2DP1* and *3DP1*). Four framework KIR genes (*2DL4*, *3DL2*, *3DL3* and *3DP1*) exist in almost all populations [4]. Fourteen functional KIR genes have been identified and confirmed, of which seven are inhibitory (KIR*2DL1*-*3*, *2DL5* and *3DL1*-*3*) and six are activating (KIR2*DS1*-*5* and *3DS1*), with only KIR*2DL4* showing both inhibitory and activating potential [1, 5, 21]. According to the numbers and types, KIR haplotypes are broadly classified into two groups: group A haplotypes have a fixed KIR gene (*2DL1*, *2DL3*-*4*, *3DL1*-*3*, *2DS4*, *2DP1* and *3DP1*), in contrast, group B haplotypes have variations in genetic content which is comprised of several genes and alleles that are not found in A haplotypes. Especially, KIR*2DS1*-*3*, *2DS5*, *2DL2*, *2DL5* and *3DS1* are associated only with group B haplotypes, thus B haplotypes generally encode more activating KIR receptors than A haplotypes which encode a single activating receptor (KIR*2DS4*). Both group A and B are present in all populations, but their frequencies vary considerably [1, 6]. Many individuals carry two copies of A haplotypes (AA genotypes). The remainders carry Bx genotypes, which are consisted of either one copy of A haplotype and one copy of B haplotype (AB genotypes) or two copies of B haplotypes (BB genotypes). Based on the presence and absence of two distinct gene clusters (C4, KIR*2DS2*-*2DL2*-*2DS3*-*2DL5*; T4, *KIR3DS1*-*2DL5*-*2DS5*-*2DS1*), Bx genotype carries are divided into four subsets: C4Tx (presence of C4 and absence of T4), CxT4 (absence of C4 and presence of T4), C4T4 (presence of both C4 and T4), and CxTx (absence of both C4 and T4) [1, 7, 8].

Usually, Group B haplotypes encode more activating KIR genes. In individuals with AA genotype, the alleles of KIR*2DS4* can encode either functional (KIR*2DS4*-*f*) or non-functional (KIR*2DS4*-*v*) variants [9, 10]. KIR*2DS4*-*v* alleles differ from the KIR*2DS4*-*f* alleles in that the former have a 22-bp deletion in exon 5, which leads to a frame shift mutation and produces a premature stop codon, preventing the formation of a functional membrane-bound receptor domain [10]. Therefore, individuals with AA genotypes which harbor KIR*2DS4*-*v* alleles will not have a membrane-bound KIR*2DS4* protein, but rather a soluble form of KIR*2DS4*. KIR*2DS4*-*f* and *2DS4*-*v* frequencies vary amongst different populations. Increasing attention has been paid to the role of KIR genetic content and allelic variation on infectious diseases such as Hepatitis C and HIV, with some studies dedicating a specific focus on KIR *2DS4* [9–14]. KIR gene diversities have been studied in many different geographical populations as previously reported [1, 9, 15–23]. There are 56 ethnic groups in China [24]. Most of the studies on KIR gene diversity have been reported on Han populations in China [18–21]. A large-scale survey on transfusion-transmitted HIV-1/2 infection among Chinese blood donors conducted by our institute showed that the positive rate for HIV infection was higher in some ethnic minorities (including data accumulated at Urumqi Blood Center, Xinjiang) than Han majority donors [25]. Expanding our understanding of the ethnic intermarriage and possibly random demographic factors could help us to determine the variation of KIR gene frequencies, which might be useful for future research on ethnicity-based diseases. Hence, in this study we chose the main minorities in Urumqi city of Northwest China, the Uyghur (comprised 10,069,346 persons [26], presented a typical mixture of Eastern and Western anthropometric traits [27]) and Kazakh (comprised 1,462,588 persons [26]), and the Tibetan (the major ethnic minority in Tibet, comprised 6,282,000 persons [26]), mostly living in Lhasa city of Southwest China, as the research objects. (Additional file 1: Figure S1).

## Methods

### Samples and DNA isolation

A total of 479 randomly selected donors were studied from three ethnic minority populations in China, including Tibetan (N = 224) volunteer blood donors in Lhasa (April 2011 to March 2012), Kazakh (N = 125) and Uyghur (N = 130) volunteer blood donors in Urumqi (March to August 2012). Informed consents of all samples used in this study were approved by the ethical Committee of Institute of Blood Transfusion. Genomic DNA was extracted from PBMC by TIANGEN blood DNA kit (Cat: #DP318-03), following the manufacturer’s instructions. The concentration of extracted DNA was adjusted to 50–100 ng/μL, and the O.D. 260/O.D. 280 ratio ranged from 1.6 to 1.9.

### KIR PCR-SSP genotyping

KIR genes were typed for the presence or absence of the 14 KIR genes, including KIR*2DL1*-*5*, *2DS1*-*5*, *3DL1*-*3* and the 2 pseudogenes (*3DP1* and *2DP1*), using multiplex PCR-SSP. In order to ensure accuracy, two different sets of primers for amplification (except *3DP1* and *2DS1*) were used while HLA-DRB1 intron amplification primers were added to the third well as a positive control. Primer designs were adopted from those previously reported by Campbell et al. [28]. The primer arrangement was referred in Table 1 PCR master-mix for the multiplex PCR-SSP which contained 3 μL of primer (10 μM) pairs, 1.5 μL of ddH_2_O, 0.5 μL of DNA, 5 μL of GoTaq green master mix (Promega, Cat: #M7123), for a total of 10 μL. The conditions for PCR cycles were as followed: five cycles at 94°C for 3 min, 94°C for 15 s, 65°C for 15 s, 72°C for 30 s; twenty-one cycles at 94°C for 15 s, 60°C for 15 s, 72°C for 30 s; four cycles at 94°C for 15 s, 55°C for 1 min, 72°C for 2 min; lastly, at 72°C for 7 min and stored at 4°C. PCR products were visualized under UV light after electrophoresis in 3% agarose gel and stained with ethidium bromide, using DL1000 DNA Marker (TIANGEN, Cat: #MD108).

**Table 1:** Multiple PCR primers combination pattern

Mix	Locus (bp)	Locus (bp)	Locus (bp)	Locus (bp)
1	2DL1 (330)	2DL2 (173)	2DL2 (151)	
2	2DS3 (190)	2DP1 (89)		
3	DRB1 (800)	3DS1 (300)	3DL1 (191)	
4	3DP1 (399/280)^a^	2DL5 (214)	2DL5 (191)	2DS1 (102)
5	2DL3 (800)	2DL4 (254)		
6	2DL3 (550)	2DS3 (242)	3DS1 (180)	
7	2DS4 (204)	3DL1 (186)	2DL1 (146)	
8	2DS4 (179/219)^b^	2DS5 (126)		
9	3DL3 (232)	2DS5 (178)	3DL2 (130)	
10	2DP1 (205)	2DS2 (175)		
11	2DS2 (240)			
12	2DL4 (288)	3DL2 (242)	3DL3 (165)	

^a^The length of 3DP1 is 399 bp and the variant 3DP1v is 280 bp.

^b^The length of 2DS4-f is 219 bp and the variant 2DS4-v is 197 bp.

### Statistical analysis

Genotypic frequency = n/N (n represented numbers of positive genotypes and N was the total number of individuals tested). The carrier frequencies (CF) of the KIR genes were determined by dividing the number of positive typing reactions by the total number of typed individuals. The estimated gene frequencies were calculated using the formula GF = 1 − (1 − CF)^1/2^. The frequencies of A and B haplotypes were calculated using the following formula: group A = 2nAA + nAB/2N and group-B = 2nBB + nAB/2N, where nAA, nAB, and nBB were the numbers of AA, AB and BB genotypes. Differences in the carrier frequencies between the studied population and other populations previously published were assessed by the standard Chi square test (x^2^) using statistical software PEMS3.1 Medicine, and p < 0.05 was considered to be statistically significant. Subsets of KIR genetic content profiles and their frequencies in populations were carried out using statistical software Graph Pad Prism5. Principal components analysis (PCA) of 22 populations [15] was performed using an R package: psych software. Genetic distances were calculated by Nei’s method using phylip [29]. On the basis of the Nei’s genetic distance, a dendrogram was constructed by the neighbor-joining method [30] and visualized with MEGA software in order to compare the frequencies of KIR genes in 22 populations [15], using bootstrap values are calculated from 100 trees.

## Results

### KIR genotypes and haplotypes

**Figure 1:**
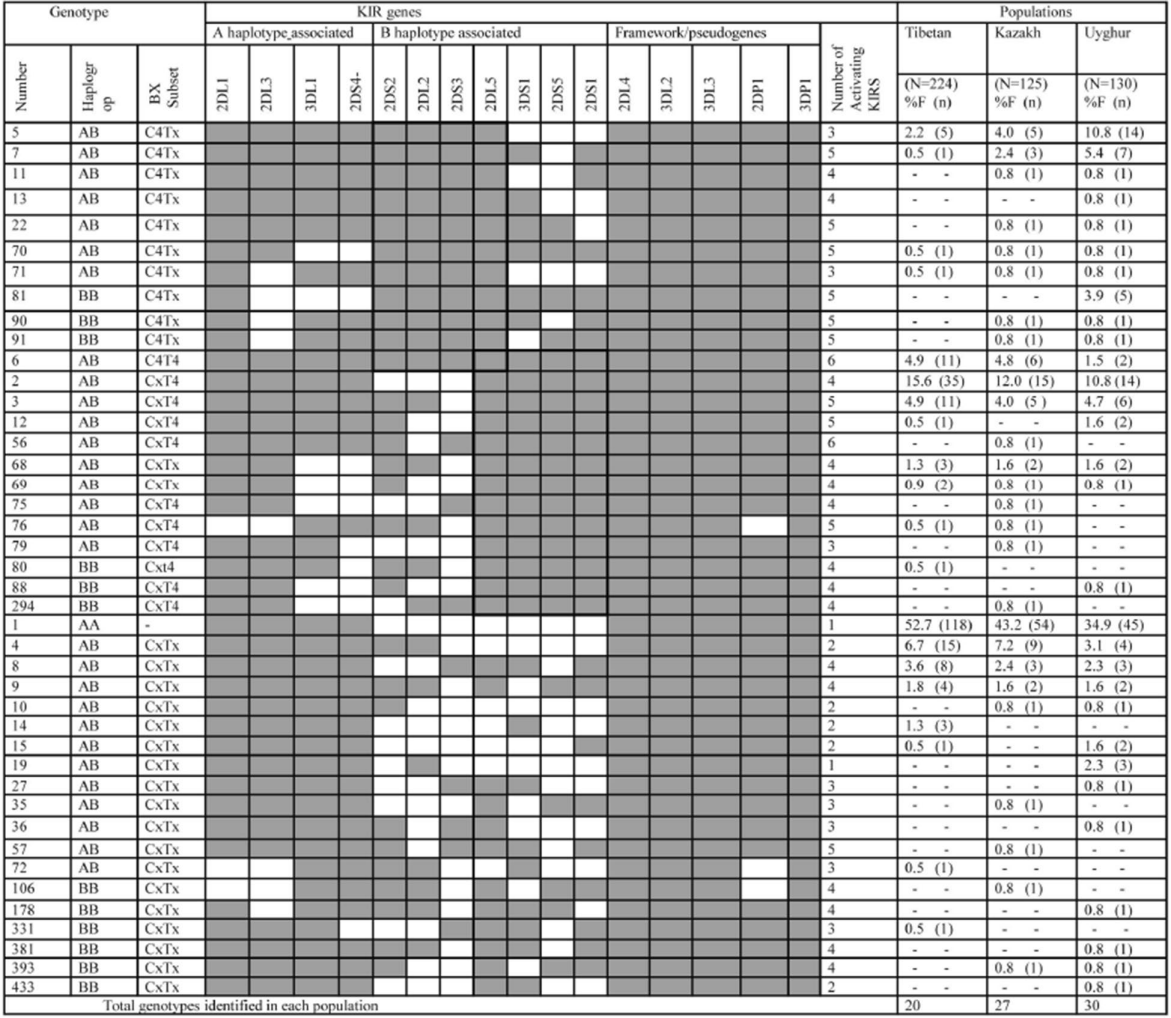
KIR gene content diversity of three Chinese populations, within 479 unrelated individuals, 42 genotypes that differed by the presence (*shaded box*) and absence (*white box*) of 16 KIR genes were detected. The frequency of each genotype is presented in percentage frequency (%F) and defined as the number of individuals carrying the genotype (n) divided by the number of individuals studied (N) in the provided population.

A total of 479 individuals, 42 genotypes were found to carry a different number and combination of 16 KIR genes (Figure 1), of which, 29 were predicted to be AB genotypes, 12 were predicted to be BB genotypes and one was AA genotype. 27 KIR genotypes were identified in Kazakhs, 30 KIR genotypes were identified in Uyghurs and 20 KIR genotypes were identified in Tibetans. The predominant genotype 1 (AA genotypes) occurred most frequently in Tibetans (52.7%, 118/224), Kazakhs (43.2%, 54/125) and Uyghurs (34.9%, 45/130). Variations in genotypes and haplotypes were significant between the Tibetan and Kazakh/Uyghur groups (Table 2): 3 displayed significant statistical difference between Tibetan and Kazakh populations, 6 displayed significant statistical difference between Tibetan and Uyghur populations, and 2 displayed significant statistical difference between Uyghur and Kazakh populations.

**Table 2:** Comparison of genotypes, haplotypes and linkage groups in Tibetan, Kazakh and Uyghur populations

Types	Tibetan	Kazakh	Uyghur	P values		
	N = 224	N = 125	N = 130	Tibetan vs Kazakh	Tibetan vs Uyghur	Kazakh vs Uyghur
	%F(n)	%F(n)	%F(n)			
AA genotypes	52.7 (118)	43.2 (54)	34.9 (45)		0.0010	
BB genotypes	0.9 (2)	4.0 (5)	9.0 (12)	0.0471	0.0001	
AB genotypes	46.4 (104)	52.8 (66)	56.1 (73)			
C4Tx genotypes	3.6 (8)	11.2 (14)	25.4 (33)	0.0049	<0.0001	0.0035
C4T4 genotypes	4.9 (11)	4.8 (6)	1.6 (2)			
CxT4 genotypes	24.1 (54)	21.6 (27)	20.0 (26)			
CxTx genotypes	14.7 (33)	15.2 (19)	16.2 (21)			
A haplogroups	75.9 (170)	69.6 (87)	63.0 (82)		0.0103	
B haplogroups	24.1 (54)	30.4 (38)	36.9 (48)		0.0103	
C4 gene-cluster	8.5 (19)	16.0 (20)	26.9 (35)	0.0326	<0.0001	0.0340
T4 gene-cluster	29.0 (65)	27.2 (34)	21.5 (28)			

The haplotype A and B were determined by using the following formula: group A = 2nAA + nAB/2N and group B = 2nBB + nAB/2N, where nAA, nAB, and nBB are the numbers of AA, AB, and BB genotypes, N = total number of population. The p values are given only for those pairwise comparisons indicating significant (P < 0.05) differences.

### KIR gene frequency

16 KIR genes were detected in Tibetans, Kazakhs and Uyghurs. The carrier frequency (CF) was listed in Table 3. Four framework genes (*2DL4*, *3DL2*, *3DL3* and pseudogene *3DP1*) were present in all individuals. KIR*2DL1*, *2DL3*, *3DL1* and *2DP1* were detected in high frequencies (>90%). Among them, pseudogene *2DP1* can be detected in all individuals of Uyghur. *KIR2DL2*, *2DL5*, *2DS1*, *2DS2*, *2DS3*, *2DS5* and *3DS1* were in low frequencies. In the Uyghurs, the frequencies of KIR*2DS4*-*f* and KIR*2DS4*-*v* were found to be 62.3% (81/130) and 60.8% (79/130); in the Kazakhs, the frequencies of KIR*2DS4*-*f* and KIR*2DS4*-*v* were found to be 64.8% (81/125) and 59.2% (74/125); in the Tibetans, the frequencies of KIR*2DS4*-*f* and *2DS4*-*v* were found to be 86.6% (194/224) and 29.5% (66/224). Four KIR genes displayed significant statistical difference between the Tibetan and Kazakh populations, eight KIR genes showed significant statistical difference between the Tibetan and Uyghur populations, while no difference was observed between the Kazakh and Uyghur populations (Table 3).

**Table 3:** Comparison of carrier frequency of KIR genes in three ethnic minority populations in China

KIR	Tibetan	Kazakh	Uyghur	P values		
	N = 224	N = 125	N = 130	Tibetan vs Kazakh	Tibetan vs Uyghur	Kazak vs Uyghur
	%CF(n)	%CF(n)	%CF(n)			
A haplotype associated						
2DL1	99.1 (222)	98.4 (123)	98.5 (128)			
2DL3	98.7 (221)	95.2 (119)	92.3 (120)		0.0056	
3DL1	97.3 (218)	95.2 (119)	91.5 (119)		0.0028	
2DS4-f	86.6 (194)	64.8 (81)	62.3 (81)	<0.0001	<0.0001	
2DS4-v	29.5 (66)	59.2 (74)	60.8 (79)	<0.0001	<0.0001	
B haplotype associated						
2DL2	24.6 (35)	33.6 (42)	43.8 (57)		0.0003	
2DL5	38.4 (86)	47.2 (59)	59.2 (77)		0.0002	
2DS1	36.2 (81)	39.2 (49)	40.8 (53)			
2DS2	25.0 (56)	36.8 (46)	46.9 (61)	0.0277	<0.0001	
2DS3	12.5 (28)	23.2 (29)	33.9 (44)	0.0146	<0.0001	
2DS5	31.3 (70)	33.6 (42)	33.1 (43)			
3DS1	35.3 (79)	36.0 (45)	39.2 (51)			
Framework/pseudogenes						
2DL4	100 (224)	100 (125)	100 (130)			
3DL2	100 (224)	100 (125)	100 (130)			
3DL3	100 (224)	100 (125)	100 (130)			
2DP1	99.1 (222)	98.4 (123)	100 (130)			
3DP1	100 (224)	100 (125)	100 (130)			

Frequency (%CF) of carriers of each gene is expressed as a percentage and defined as the number of donors carrying the gene (n) divided by the number of donors studied (N) in the given populations. The p values are given only for those pairwise comparisons indicating significant (P < 0.05) difference.

### Genetic relationships between populations

**Figure 2:**
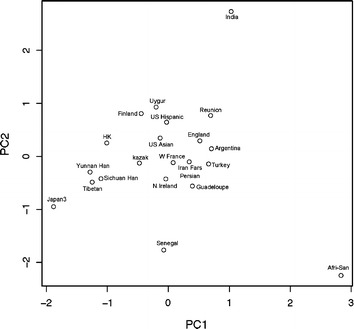
Principal component analysis (PCA) was conducted on the carrier frequencies of nine variable KIR genes. The PCA graph which was dependent on the frequencies of carriers with nine variably KIR genes (*2DL1*-*3*, *2DS1*-*4*, *3DL1* and *3DS1*) showed a worldwide perspective on the relationship between the three groups studied in this paper and other populations. PC1 and PC2 of 22 populations were given in Additional file 3: Table S2.

**Figure 3:**
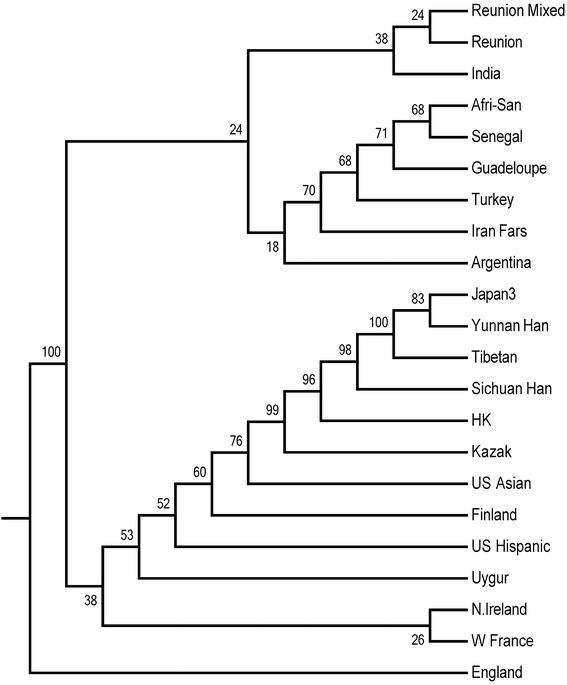
The neighbor-joining dendrogram represented the genetic distances and was created on the basis of the frequencies of 12 KIR gene (KIR*2DL1*-*3*, *2DL5*, *3DL1*, *2DS1*-*5*, *3DS1* and *2DP1*) in order to show the genetic relationship between the three groups and other previously reported populations. The KIR gene frequency of populations was given in Additional file 2: Table S1.

The PCA was generated depending on the basis of KIR gene frequencies of the three groups with other previously studied populations (as shown in Additional file 2: Table S1), PC1 and PC2 of 22 populations were given in Additional file 3: Table S2. Different geographic clusters of Asian and European were noted on the PCA plot. The most divergent were the clusters of African, Senegalese and Indian, as they did not cluster in a single group (Figure 2). Tibetans was plotted within the Asian group, the Uyghur was plotted within the European group, while the Kazakh was mapped between Asian and European groups. The neighbor-joining dendrogram was constructed, using the allelic frequency data of 12 KIR loci (KIR*2DL1*-*3*, *2DL5*, *3DL1*, *2DS1*-*5*, *3DS1* and *2DP1*) to show a relationship between the three groups and other previously reported populations [15] (Additional file 2: Table S1). In this dendrogram, there were four main clusters (Figure 3): European, African, Asian and Indian cluster. The Uyghur population was located in the European cluster, the Tibetan was located in the Asian cluster, while the Kazakh was located between European and Asian clusters. Genetic distance was shown in Figure 3.

### Genetic relationships between three ethnic populations centromeric and telomeric clusters of KIR genes (CxTx)

**Figure 4:**
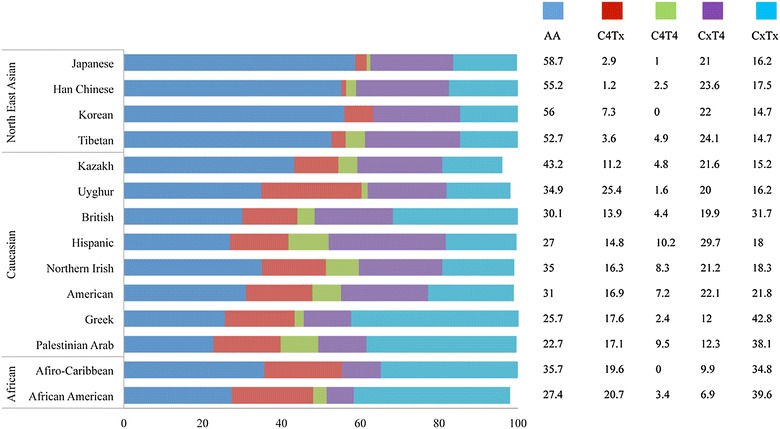
Subsets of KIR genetic content profiles and their frequency in populations. The frequencies of unique KIR genotype subsets in Tibetan, Kazakh and Uyghur populations were elucidated.

We found that the three groups carried all four subsets of Bx genotypes (CxT4, C4Tx, C4T4, and CxTx, Figure 1). 55 with the expression of C4Tx genotypes carried three to five activating KIR genes, among them, 49.1% (27/55) carried three activating KIRs and 45.3% (25/55) carried five activating KIRs. 108 with the expression of CxT4 genotypes carried three to six activating KIR genes, among them, 73.2% (79/108) carried four activating KIRs. 1 with the expression of C4T4 genotypes carried six activating KIRs. 73 individuals displaying CxTx carried one to five activating KIRs, among them, 50.7% (37/73) carried two activating KIRs, while 37.0% (27/73) carried four activating KIRs (Figure 1). The Uyghurs (25.4%, 33/130) comprised higher frequencies of Bx genotypes with C4Tx than the Kazakhs (11.2%, 14/125) and Tibetans (3.6%, 8/224, Figure 4).

## Discussion

KIR genotypes show extensive variation in populations according to different geographical regions and different ethnic groups, due to the presence or absence of different KIRs in individual haplotypes and allelic polymorphism of KIR genes [17]. In this study, Tibetans were detected to display a lower level of genotypic diversity, with the predominant genotypes *ID1* (AA genotypes) [15] and *ID2.* The cumulative frequency of the top five genotypes 1–4, 6 was 84.8% (190/224), which showed that KIR genotype distribution in Tibetan populations was relatively concentrated. Meantime, Uyghurs and Kazakhs displayed a higher genotypic diversity, with the predominant genotypes *ID1*. Intriguingly, Uyghur displayed consistency with Caucasians on the level of KIR allelic frequency [9], which comprised high frequencies of Bx genotypes (absence of *KIR3DS1*-*2DL5*-*2DS5*-*2DS1*), while Tibetans shared a lower frequencies consistent with Northeast Asian (Han Chinese [21], Korean [22] and Japanese [23]). Our data on Kazakhs showed that the KIR allelic frequency is between that of Caucasians and Northeast Asians. Many studies on KIR gene diversity have been reported on Han populations in China [18–21]. Expanding our understanding of ethnic intermarriage and possibly random demographic factors for ethnic minorities could help us to determine the variation of KIR gene frequencies, which might be useful for future research on ethnicity-based diseases.

Activating KIR genes were found to show greater variability in frequencies than inhibitory KIR genes, which was similar to those previously reported in other populations [1]. KIR*2DS4* is the only activating gene in AA genotypes, with the other 5 activating genes (KIR*2DS1*-*3*, *2DS5* and *3DS1*) being absent [5, 21]. In this study, KIR*2DS4*-*v* was found to have a higher frequency in Uyghurs and Kazakhs, which was consistent with the frequency in Caucasians [9]. Tibetans had a lower KIR*2DS4*-*v* frequency when compared with Kazakhs and Uyghurs, more similar to Asian populations (Han Chinese, Japanese and Koreans) [21–23].

The PCA and the generated dendrogram aligned with the origins of ancestry distributing across vast geographic regions. In our data, the Uyghur was located in the European cluster, showing a similarity to the origins of European Caucasian ancestry. This was consistent with previously report that Uyghur population, settled in Xinjiang, was a population presenting a typical admixture of Eastern and Western anthropometric traits [25]. The Kazakh was between Eastern Asian and European Caucasian groups, demonstrating an array of mixed anthropological features of Europeans and Asians. This could help to further understand the origins of ancestry, the pattern of human migration, the intermarriage between ethnicities and the genetic distances between different populations or ethnic groups.

## Conclusions

The KIR gene frequencies of the Tibetan and Uyghur populations were consistent with the geographic and ethnic distribution, while the Kazakhs showed unique genetic content and allelic polymorphisms. Better understanding the ethnic intermarriage and possibly random demographic factors could help determine the factors in the variation of KIR gene frequencies, which could be useful for future research on ethnicity-based diseases.

## Additional files

Additional file 1:
**Figure S1.** Map of China showing the city of three study populations. DNA samples of the Kazakh and Uyghur ethnic minority populations were collected from the Xinjiang autonomous region (Urumqi) of Northwest China, the Tibetan ethnic minority populations were collected from the Tibet autonomous region (Lhasa) of Southwest China. Additional file 2:
**Table S1.**  KIR diversity in three ethnic minority populations in China with other populations. Additional file 3:
**Table S2.** PC1 and PC2 of 22 populations.
