# Supplementary figures and images for: KIR diversity in three ethnic minority populations in China
Source: J Transl Med. 2015 Jul 11;13:221. doi: 10.1186/s12967-015-0544-7 (PMC4498514; doi:10.1186/s12967-015-0544-7)

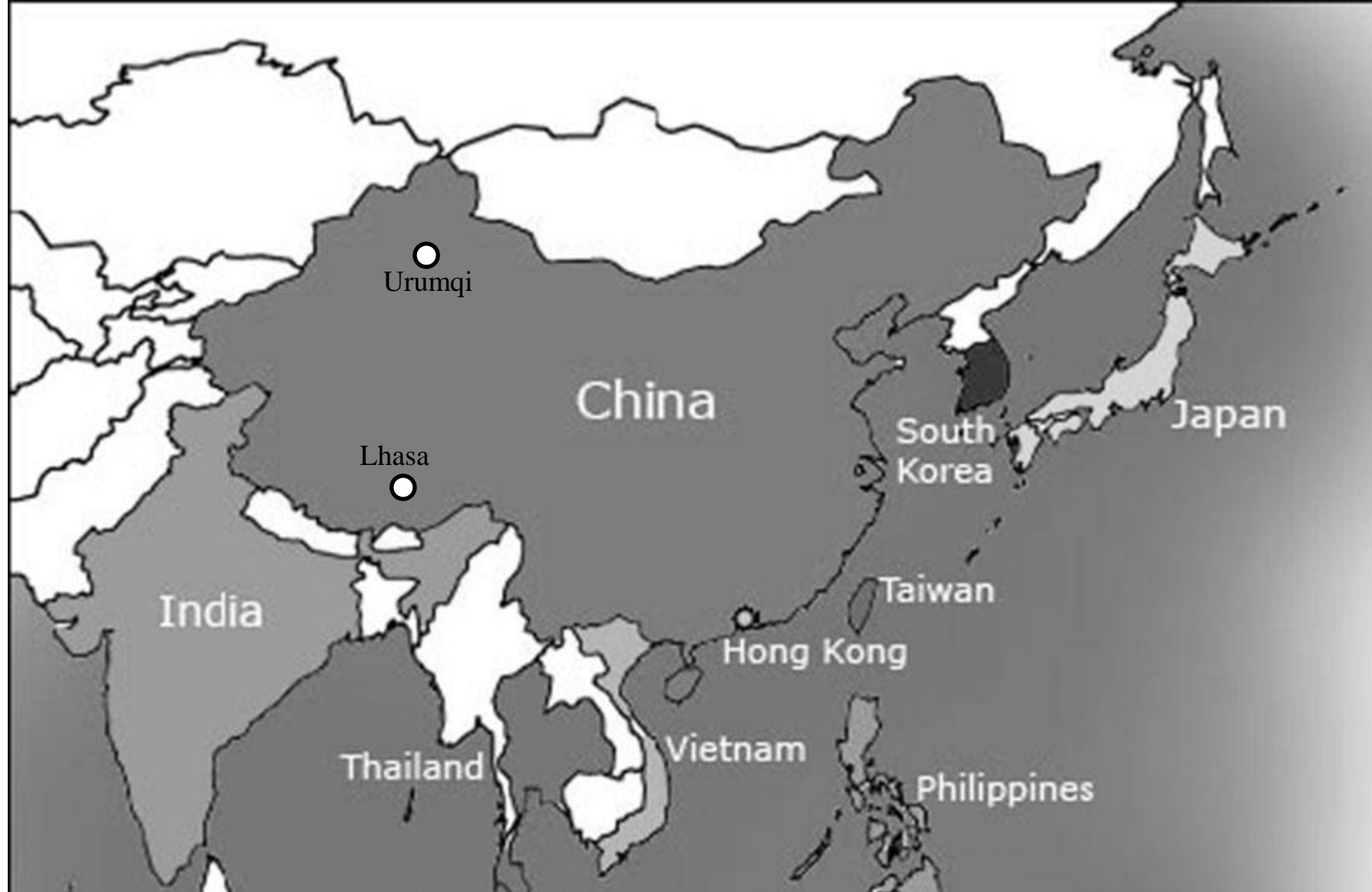

Supplement: Additional file 1: — Figure S1. Map of China showing the city of three study populations. DNA samples of the Kazakh and Uyghur ethnic minority populations were collected from the Xinjiang autonomous region (Urumqi) of Northwest China, the Tibetan ethnic minority populations were collected from the Tibet autonomous region (Lhasa) of Southwest China. [file 12967_2015_544_MOESM1_ESM.pdf]
