# Supplementary material for: KIR diversity in three ethnic minority populations in China
Source: J Transl Med. 2015 Jul 11;13:221. doi: 10.1186/s12967-015-0544-7 (PMC4498514; doi:10.1186/s12967-015-0544-7)
Supplement: Additional file 2: — Table S1. KIR diversity in three ethnic minority populations in China with other populations. [file 12967_2015_544_MOESM2_ESM.doc]

| Population/Gene | N | 2DL1 | 2DL2 | 2DL3 | 2DL5 | 2DP1 | 2DS1 | 2DS2 | 2DS3 | 2DS4 | 2DS5 | 3DL1 | 3DS1 |
| --- | --- | --- | --- | --- | --- | --- | --- | --- | --- | --- | --- | --- | --- |
| Argentina | 365 | 96.1 | 62.5 | 86.5 | 55.7 | 96.1 | 45.4 | 54.5 | 28.6 | 95 | 35.4 | 95 | 42 |
| Finland | 101 | 100 | 41.6 | 96 | 55.4 | 100 | 48.5 | 41.6 | 23.8 | 94.1 | 45.5 | 93.1 | 49.5 |
| W France | 108 | 97 | 50 | 91 | 47 | 97 | 36 | 51 | 31 | 96 | 27 | 96 | 44 |
| Guadeloupe | 118 | 100 | 61 | 84 | 61 | 100 | 38 | 58 | 33 | 98 | 32 | 99 | 24 |
| HK | 100 | 99 | 28 | 98 | 45 | 99 | 40 | 28 | 25 | 94 | 26 | 94 | 39 |
| N.Ireland | 154 | 97.9 | 47.4 | 90.3 | 50 | 98.1 | 38.3 | 47.4 | 28.2 | 98.1 | 31.2 | 98.1 | 39 |
| Japan3 | 132 | 100 | 11.4 | 100 | 35.6 | 100 | 33.3 | 11.4 | 13.6 | 99.2 | 22 | 99.2 | 31.9 |
| Reunion | 101 | 96 | 55 | 91 | 58 | 96 | 42 | 53 | 38 | 91 | 36 | 92 | 42 |
| Senegal | 118 | 100 | 55 | 90 | 52 | 100 | 13 | 42 | 24 | 100 | 30 | 99 | 4 |
| Afri-San | 91 | 95.6 | 73.6 | 60.4 | 68.1 | 92.3 | 14.3 | 72.5 | 25.3 | 100 | 63.7 | 98.9 | 2.2 |
| US Asian | 150 | 96.7 | 40 | 90.7 | 55.3 | 99.3 | 43.3 | 37.3 | 27.3 | 95.3 | 36.7 | 94 | 44 |
| US Hispanic | 128 | 98.4 | 46.9 | 93.8 | 60.2 | 100 | 44.5 | 46.9 | 27.3 | 93.8 | 43 | 93.8 | 45.3 |
| India | 145 | 99.3 | 62.8 | 81.9 | 71 | 99.3 | 62.8 | 62.8 | 53.8 | 86.1 | 51 | 87.4 | 62.2 |
| Reunion Mixed | 101 | 96 | 55 | 91 | 58 | 96 | 42 | 53 | 38 | 91 | 36 | 92 | 42 |
| Iran Fars Persian | 248 | 98 | 56.8 | 91 | 58.5 | 98 | 35 | 54 | 37.9 | 96 | 25.4 | 96 | 33 |
| England | 584 | 96.2 | 52.9 | 89.7 | 55.5 | 96.2 | 43.6 | 53.4 | 29.5 | 94.3 | 36.3 | 94.4 | 44.4 |
| Turkey | 154 | 98.1 | 59.7 | 85.7 | 55.2 | 96.1 | 36.4 | 60.4 | 35.1 | 94.2 | 28.6 | 96.1 | 32.5 |
| ***Uygur*** | ***130*** | ***100*** | ***43.9*** | ***92.3*** | ***59.2*** | ***100*** | ***42.3*** | ***47.7*** | ***33.9*** | ***91.5*** | ***32.3*** | ***91.5*** | ***40*** |
| ***Kazak*** | ***125*** | ***98*** | ***34*** | ***95*** | ***47*** | ***98*** | ***39*** | ***37*** | ***23*** | ***96*** | ***34*** | ***95*** | ***36*** |
| ***Tibetan*** | ***224*** | ***99*** | ***25*** | ***99*** | ***38*** | ***99*** | ***36*** | ***25*** | ***13*** | ***96*** | ***31*** | ***97*** | ***35*** |
| *Sichuan Han* | *286* | *99* | *25* | *98* | *43* | *99* | *37* | *28* | *18* | *97* | *28* | *97* | *36* |
| *Yunnan Han* | *150* | *99* | *25* | *99* | *37* | *99* | *36* | *25* | *15* | *95* | *27* | *95* | *32* |

***Table S1* KIR diversity in three ethnic minority populations in China with other populations** ^[15]^
