# Supplementary material for: KIR diversity in three ethnic minority populations in China
Source: J Transl Med. 2015 Jul 11;13:221. doi: 10.1186/s12967-015-0544-7 (PMC4498514; doi:10.1186/s12967-015-0544-7)
Supplement: Additional file 3: — Table S2. PC1 and PC2 of 22 populations. [file 12967_2015_544_MOESM3_ESM.docx]

**Table S2 PC1 and PC2 of 22 populations**

| Population | PC1 | PC2 |
| --- | --- | --- |
|  |  |  |
| Argentina | 0.705845563 | 0.141755776 |
| Finland | -0.445649239 | 0.805179765 |
| W France | 0.078457904 | -0.120552064 |
| Guadeloupe | 0.393757989 | -0.56246762 |
| HK | -1.010371546 | 0.250211543 |
| N. Ireland | -0.042546775 | -0.427616634 |
| Japan3 | -1.883269922 | -0.951762278 |
| Reunion | 0.692399385 | 0.768129991 |
| Senegal | -0.076200413 | -1.768419077 |
| Afri-San | 2.830602831 | -2.248918451 |
| US Asian | -0.135096491 | 0.343497916 |
| US Hispanic | -0.029510676 | 0.642416084 |
| India | 1.029709036 | 2.730486134 |
| Reunion Mixed | 0.692399385 | 0.768129991 |
| Iran Fars Persian | 0.341458887 | -0.104635261 |
| England | 0.515857365 | 0.290839021 |
| Turkey | 0.658773711 | -0.14518451 |
| ***Uygur*** | **-0.201978498** | **0.926918005** |
| ***Kazak*** | **-0.475291629** | **-0.129017037** |
| ***Tibetan*** | **-1.249530643** | **-0.487857272** |
| *Sichuan Han* | -1.105703012 | -0.42255476 |
| *Yunnan Han* | -1.284113213 | -0.298579262 |
